# The impact of financial incentives on the implementation of asthma or diabetes self-management: A systematic review

**DOI:** 10.1371/journal.pone.0187478

**Published:** 2017-11-06

**Authors:** Tracy Jackson, Michael D. Shields, Liam G. Heaney, Marilyn Kendall, Christina J. Pearce, Chi Yan Hui, Hilary Pinnock

**Affiliations:** 1 Asthma UK Centre for Applied Research, Usher Institute of Population Health Sciences and Informatics, The University of Edinburgh, Edinburgh, United Kingdom; 2 Asthma UK Centre for Applied Research, Centre for Experimental Medicine, Queen’s University Belfast, Belfast, United Kingdom; 3 Usher Institute of Population Health Sciences and Informatics, The University of Edinburgh, Edinburgh, United Kingdom; 4 UCL School of Pharmacy, Centre for Behavioural Medicine, BMA House, London, United Kingdom; Teesside University, UNITED KINGDOM

## Abstract

**Introduction:**

Financial incentives are utilised in healthcare systems in a number of countries to improve quality of care delivered to patients by rewarding practices or practitioners for achieving set targets.

**Objectives:**

To systematically review the evidence investigating the impact of financial incentives for implementation of supported self-management on quality of care including: organisational process outcomes, individual behavioural outcomes, and health outcomes for individuals with asthma or diabetes; both conditions with an extensive evidence base for self-management.

**Methods:**

We followed Cochrane methodology, using a PICOS search strategy to search eight databases in November 2015 (updated May 2017) including a broad range of implementation methodologies. Studies were weighted by robustness of methodology, number of participants and the quality score. We used narrative synthesis due to heterogeneity of studies.

**Results:**

We identified 2,541 articles; 12 met our inclusion criteria. The articles were from the US (n = 7), UK (n = 4) and Canada (n = 1). Measured outcomes were HbA1c tests undertaken and/or the level achieved (n = 10), written action plans for asthma (n = 1) and hospital/emergency department visits (n = 1). Three of the studies were part of a larger incentive scheme including many conditions; one focused on asthma; eight focussed on diabetes. In asthma, the proportion receiving ‘perfect care’ (including providing a written action plan) increased from 4% to 88% in one study, and there were fewer hospitalisations/emergency department visits in another study. Across the diabetes studies, quality-of-care/GP performance scores improved in three, were unchanged in six and deteriorated in one.

**Conclusions:**

Results for the impact of financial incentives for the implementation of self-management were mixed. The evidence in diabetes suggests no consistent impact on diabetic control. There was evidence from a single study of improved process and health outcomes in asthma. Further research is needed to confirm these findings and understand the process by which financial incentives may impact (or not) on care.

**Trial registration:**

**Protocol registration number:** CRD42016027411

## Introduction

The prevalence of long term conditions is increasing and supported self-management is a strategy to enable healthcare services to cope with this increase [1]. Self-management has been defined as ‘the tasks that individuals must undertake to live with one or more chronic conditions. These tasks include having the confidence to deal with medical management, role management and emotional management of their conditions’ [2]. A core responsibility of professionals and healthcare organisations is to provide support to enable people with long-term conditions to manage their own condition [3]. Asthma and diabetes are two long-term conditions with a robust evidence base for supported self-management [4, 5].

### Asthma and diabetes in the United Kingdom

In the United Kingdom (UK), 3.6 million people are being treated for clinician-diagnosed asthma, costing £1.1 billion annually in healthcare resources [6]; globally up to 334 million people are affected [7]. Supported self-management, including education and personalised asthma action plans (PAAPs) have consistently been shown to improve asthma control, minimise exacerbations and reduce emergency use of healthcare resources [5, 8–10]. The British Guideline on the Management of Asthma recommends that all individuals with asthma should be provided with self-management education and offered a PAAP [11]. However, ownership of PAAPs remains low with only 24% of people with asthma in the UK reporting that they are in possession of a PAAP [12].

There are 3.5 million people in the UK who have been diagnosed with diabetes and approximately 1.1 million are as yet undiagnosed [13]. Self-management of diabetes, including lifestyle changes, adherence to medication, monitoring blood sugars and adjusting dosages accordingly can greatly improve quality of life. However, only 16% of individuals with diabetes were offered an education course when first diagnosed [14]. In the UK GPs are incentivised through the Quality Outcome Framework (QOF) to provide eight routine checks, as recommended by National Institute for Health and Care Excellence, to individuals with diabetes [15] but just 43.3% of individuals with Type 1 diabetes were receiving all eight of the recommended care processes in 2010–11 and this has further dropped to 36.5% in 2015–16 [16].

### Financial incentive schemes

Physician focussed financial incentive schemes are a potential strategy for changing physician behaviour to improve quality of care which has recently been employed in national and local schemes [15, 17]. Financial incentives which place the physician at financial risk have been found to modify resource use (measured by hospitalisation rates and primary care visits) but the evidence investigating the impact of bonus payments shows mixed results in terms of impact [18]. Pay-for-performance can be effective in healthcare but providers should be involved with programme design and schemes need to be tailored to the setting [19] and patient population [20]. Incentives need to be tied to improvements in information systems and quality reporting standards [21]. Policy makers must remain aware of unintended consequences and carefully weigh potential benefits against the risks for their individual setting [22]. For example, general practitioners (GPs) interviewed about QOF highlighted the potential for reduced continuity of care, lack of attention to non-incentivised conditions and potential damage to healthcare professional’s internal motivation [23].

There is therefore uncertainty about the appropriate models employed in financial incentive schemes and inconclusive insufficient evidence of their impact on quality of care [24]. This paper reports the findings of a systematic review describing the features of financial incentive schemes promoting supported self-management, and evaluating the impact of the schemes on quality of care, specifically organisational process outcomes, individual behavioural outcomes, and health outcomes.

## Methods

Our protocol is available on PROSPERO, registration number: CRD42016027411 (S1 Appendix), and we followed the procedures described in the Cochrane Handbook for Systematic Reviews of Interventions [25]. The searches were run in November 2015 and updated in May 2017.

### Search strategy

Our PICOS search strategy is shown in Table 1. We searched eight databases: Cochrane Central Register of Controlled Trials (CENTRAL); Cochrane Database of Systematic Reviews (CDSR); MEDLINE; PsychInfo; CINAHL; ScienceDirect; Web of Science; Embase. We searched for asthma OR diabetes AND financial incentives AND self-management keywords (S2 Appendix gives a detailed search strategy) and did not restrict the date range. The bibliographies of all eligible studies were examined to identify potential studies for inclusion and we searched registries for studies in progress.

**Table 1:** PICOS search strategy.

COMPONENT	DESCRIPTION, INCLUSION/EXCLUSION CRITERIA
POPULATION	• Healthcare professionals incentivised (or whose organisation is incentivised) to provide self-management • Individuals with asthma or diabetes receiving care from an organisation which is receiving financial incentives
INTERVENTION	• Any financial incentive provided to a healthcare organisation and/or healthcare professionals that is designed to improve supported self-management in asthma or diabetes
COMPARISON	• Healthcare professionals not incentivised (or whose organisation is not incentivised) to provide self-management. • Individuals with asthma or diabetes who are receiving usual, non-incentivised care
OUTCOMES	• Organisational process: increase in quality of care, PAAP ownership and/or asthma/diabetes reviews • Disease control: decrease in exacerbations and/or hospitalisations, improved asthma/diabetes control • Individual behaviour: self-efficacy, activation, adherence to medication
SETTING	• Any healthcare setting
STUDY DESIGN	• Randomised controlled trials (RCTs) • Quasi -RCTs • Controlled before and after studies • Interrupted time series • Repeated measures

### Study selection

**Fig 1:**
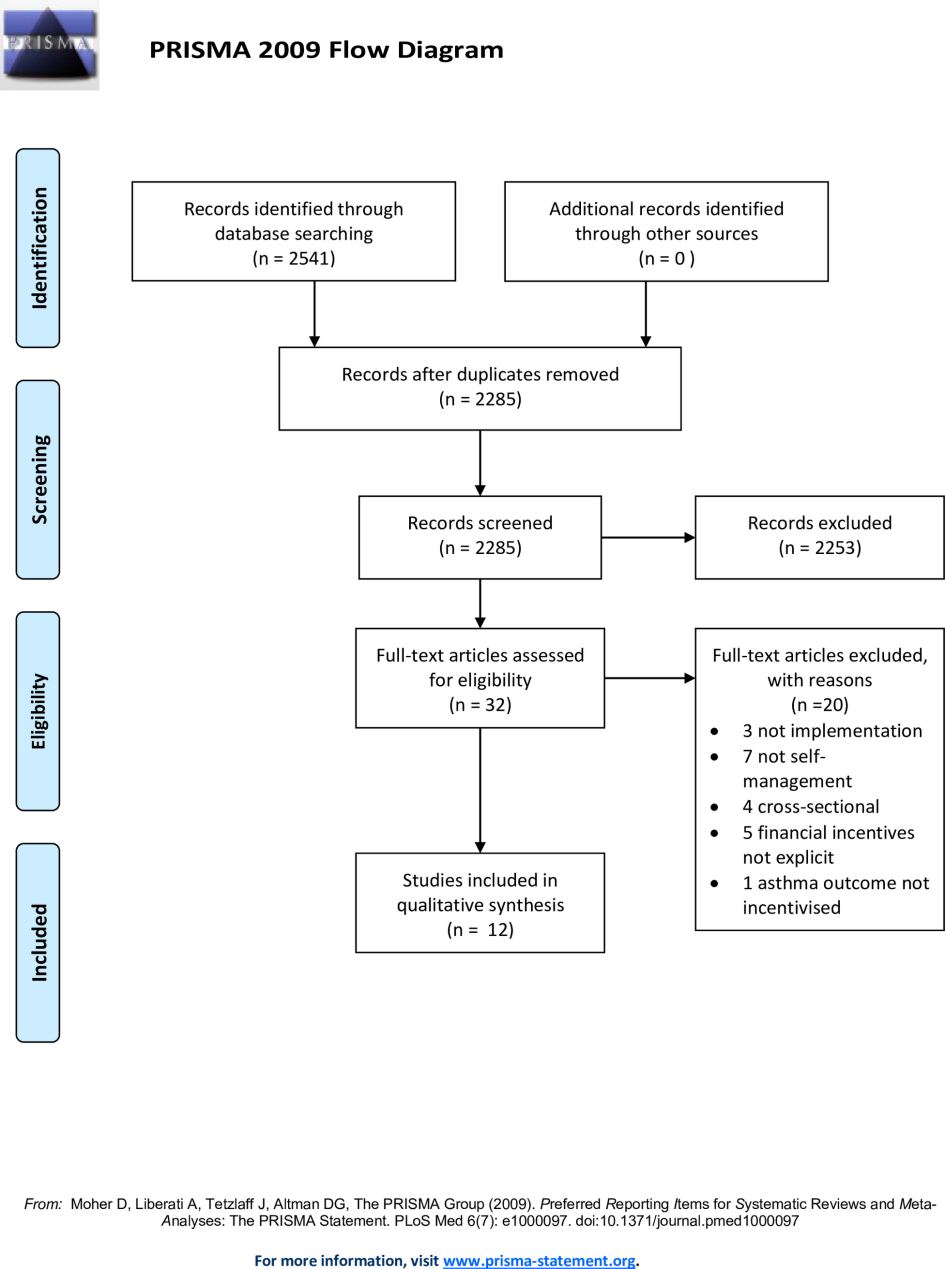
Prisma 2009 flow diagram.

One reviewer (TJ) conducted the searches in November 2015 and May 2017, 2,541 articles were identified (Fig 1). Two reviewers (TJ and HP) screened a random selection of 100 papers, compared and discussed decisions in order to reach agreement on the application of the inclusion/exclusion criteria. After this training process, one reviewer (TJ) screened the remaining titles and abstracts for potentially relevant papers. Full text screening was undertaken by two reviewers (TJ and CYH) independently. Uncertainties and disagreements were resolved in discussion with a third reviewer (HP). Reviewers achieved 100% agreement with articles selected for inclusion.

### Quality assessment

It was anticipated that most of the studies included would be reporting implementation studies, therefore we did not apply any methodological filters. In order to weight the papers which we expected would include a diverse range of methodologies, we adopted the approach of Pinnock 2015 [4] and papers were classified by robustness of study design, number of participants and the quality score (using Downs’ and Black checklist [26]).

### Outcomes

We defined our outcomes of interest in three categories:

1. *Organisational process outcomes*. Specific examples are ownership of a personalised asthma action plan; attendance at patient training self-management courses for asthma or diabetes; attendance at reviews providing supported self-management of asthma or diabetes.
2. *Individual behavioural outcomes*. Examples include self-efficacy, activation, adherence to preventer medication, adherence to insulin regimes.
3. *Health outcomes*. Examples are symptom control, reducing asthma exacerbations, unscheduled care or use of emergency health services, and measuring glycaemic control for people with diabetes (glycated haemoglobin (HbA1c) levels reflect the overall glycaemic exposure over the previous 2–3 months) [27].

### Data extraction

Data were extracted from included papers by two reviewers (TJ and CJP) using a previously piloted customised version of the Effective Practice and Organisation of Care (EPOC) Good Practice data extraction form [28]. The extracted data were then compared and disagreements resolved by discussion. We extracted details about the interventions under the following headings: “setting”, “risk of bias assessment”, “participants”,”intervention groups” “methods”, “outcomes” and “results”.

Linked papers of the included studies were checked for descriptions of interventions, nested qualitative studies, and process evaluations in order to supplement the information available and to provide context. All authors were contacted to ask if any follow up work had been conducted on their study and if they had any data available that were not included in the systematic review.

### Analysis and synthesis

We anticipated substantial heterogeneity in study design and intervention [29]; meta-analysis was therefore not appropriate and a narrative synthesis was undertaken. Asthma and diabetes papers were analysed separately. We classified components of the interventions (e.g. whether the financial incentive was paid to the individual (self-employed) healthcare professional or to an organisation); whether payment was for achieving process standards (e.g. attendance at a diabetes course) or health outcomes (e.g. reduced unscheduled care). A matrix of interventions was developed with the interventions being shown to be effective or ineffective under the headings of: “organisational process”; “measure of disease control” and “individual behaviour”. We used Adams’ 2013 framework, which has been specifically designed for documenting financial incentive interventions [30]. The framework contains nine domains which we used to identify the features and describe the schemes in detail. These domains are: direction (positive reward or avoidance of penalty), form (cash or healthcare costs), magnitude (total value of incentive available to participant), certainty (certainty of receiving payment if behaviour is successfully changed: certain, certain chance or uncertain chance), target behaviour (process, intermediate or outcome), frequency of reward (all or some instances incentivised), immediacy (time between behaviour and payment), schedule (fixed or variable), and recipient(s) of incentives (clinicians).

We synthesised our results in the form of Harvest Plots, as they are a useful method for illustrating the different effects of interventions because they represent all relevant data in one plot [31]. In a Harvest plot, each bar represents an individual study, the bar colour indicates the study design, the bar height reflects the number of participants in the study and the number reflects the Downs and Black quality score.

## Results

From the 2,541 papers found, 12 papers were eligible for the systematic review (Fig 1 is the PRISMA diagram with details of the selection process).

### Study characteristics

The 12 papers were published between 2004–2017, seven were conducted in the United States of America [32–38], four in the UK [39–42] and one in Canada [43]. One study reported on an asthma-only scheme [36], three focused on diabetes-only schemes [32, 33, 43] and the remaining eight looked at diabetes within a multiple condition scheme [34, 35, 37–42].

### Risk of bias of included studies

There were problems with allocation concealment, selection bias, purposive sampling, random sequence generation and selective outcome reporting in the selected studies. We used Review Manager 5.3 to record and generate the risk of bias summary figure for the included studies (S1 Fig). Beck’s [32] participants had volunteered to take part in the intensive case management programme and the control group were those who had chosen not to, creating a non-randomised, biased sample biased by willingness to participate. Conrad’s [34] participating group were selected by the Health Insurer, Fagan’s [35] participants were selected by the managed care organisation as they had a “leadership which was willing to champion the proposed quality improvement initiative” and Gulliford’s [39] participants were a self-selected group that agreed to participate in an evaluation of diabetes care. Due to the nature of financial incentive schemes, participant blinding is not an option so that allocation concealment was an important source of bias.

### Study quality and weight of evidence

**Fig 2:**
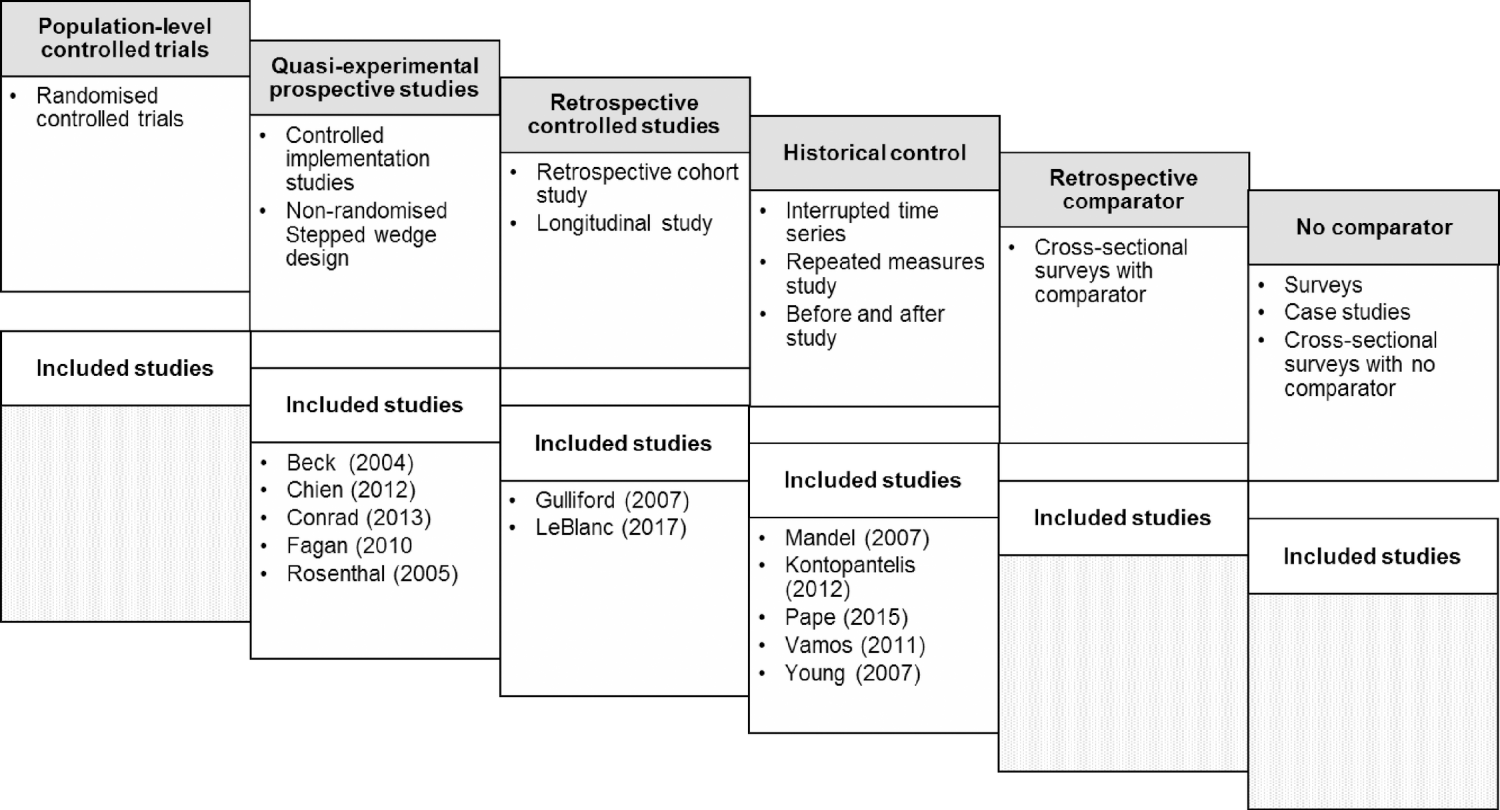
Hierarchy of included studies. Hierarchy based on: randomisation and status of comparator groups; prospective/retrospective design. These categories overlap and other factors will influence the robustness of the evidence (adapted for this review from Pinnock 2015 [4]).

The study designs varied (Fig 2) and included: five quasi-experimental [32–35, 37]; three interrupted time series [38, 40, 42]; two longitudinal [39, 43]; one repeated measures [36] and one controlled before and after study [41].

The quality scores ranged from 10 to 18 (Table 2). In common with other reviews assessing the quality of implementation studies [44], we observed that some questions in the Downs’ and Black checklist [26] were not applicable to studies involving financial incentives. For example, blinding of participants is not relevant in schemes which rely on publicity to promote financial incentives awarded for achieving pre-set targets. Similarly, questions regarding the randomisation process were not applicable to many of the quasi-experimental studies.

**Table 2:** Characteristics of included studies.

Author, date, country, LTC, intervention length	Design, participants and quality	Intervention (domains of financial incentives framework*) Recipient = clinician for all studies	Comparison group	Results (all statistics details given where available)	Impact
Beck (2004) USA Diabetes 1–15 months	Quasi-experimental. 1 hospital, 16 paediatric patients who had an incident of diabetic ketoacidosis. Quality score = 15	Pediatric Diabetes Intensive Case Management • Direction: avoidance of penalty • Form: healthcare costs • Magnitude: variable • Chance: uncertain • Target: process • Frequency: all instances incentivised • Immediacy: unclear • Schedule: variable	Participants who opted out of intervention	**Organisational process** Programme participation • Participants greater telephone contact (16 crisis management calls vs 0; p = 0.001) **Disease control** Hospital admissions • Decrease in hospital admissions from intervention group (1 emergency department visit or diabetic ketoacidosis episode vs 5 diabetic ketoacidosis hospitalisations; p = 0.039)	Positive for all outcomes
Chien (2012) USA Diabetes 5 years	Quasi-experimental. 118 practices, 5557 patients with diabetes Quality score = 13	Hudson Health Plan P4P program • Direction: positive reward • Form: cash • Magnitude: % of fee schedule • Chance: certain • Target: process • Frequency: all instances incentivised • Immediacy: annually • Schedule: fixed	Medicaid-focused health plans within New York	**Organisational process** HBa1C testing Intervention group • HbA1c testing: 2003 = 84% & 2004 = 85%, 2006 = 86% & 2007 = 91% Control Group • HbA1c testing: 2003 = 83% & 2004 = 85%, 2006 = 86% & 2007 = 87% **Disease control** HbA1c levels Intervention group • HbA1c <9b: 2003 = 36% & 2004 = 35%, 2006 = NA & 2007 = 32% Control group • HbA1c <9b: 2003 = 43% & 2004 = 38%, 2006 = NA & 2007 = 33% (The coefficient on intervention*post (difference in difference) was reported as not significant in these results, no p value provided.)	No significant effect on either outcome
Conrad (2013) USA Diabetes 4 years	Quasi-experimental. 19 medical groups, 21,365 patients Quality score = 10	Washington state P4P scheme • Direction: positive reward • Form: cash • Magnitude: % of revenue • Chance: certain • Target: process • Frequency: some instances incentivised • Immediacy: annually • Schedule: variable	5 Medical groups not part of the QSC or QIP (not randomised)	**Organisational process** HbA1c testing Quality Incentive Programme • regression results: 2003–04 = -0.001 & 2005–07 = -0.04 Quality scorecard • regression results: 2003–04 = -0.019 & 2005–07 = -0.004	Negative
Fagan (2010) USA Diabetes 12 months	Quasi-experimental. 20,943 65+ year old patients. Quality score = 16	Chronic care improvement initiative consisting of P4P practice-based care co-ordination • Direction: positive reward • Form: cash • Magnitude: % of capitation fee • Chance: certain • Target: process • Frequency: some instances incentivised • Immediacy: annually • Schedule: variable	No financial incentive but retained a call centre disease management program	**Organisational process** HbA1c testing • Intervention Group–Odds ratio = 1.66; 95%CI (1.14, 2.43) • Comparison Group–Odds ratio = 3.76; 95%CI (3.42, 4.13) • Intervention relative to Comparison–Odds ratio = 0.44; 95%CI (0.30, 0.65)	No effect
Gulliford (2007) UK Diabetes 12 months	Longitudinal. 26 general practices, 2099 patients. Quality score = 17	Quality Outcome Framework (QOF) • Direction: positive reward • Form: cash • Magnitude: Set £ value per point • Chance: certain • Target: process • Frequency: some instances incentivised • Immediacy: annually • Schedule: variable	Pre QOF	**Organisational process** HbA1c testing • HbA1c recorded in year (mean): 2000 = 60, 2001 = 72, 2002 = 80, 2003 = 78, 2005 = 95 **Disease control** HbA1c levels • HbA1c ≤7.4% (mean): 2000 = 22, 2001 = 32, 2002 = 37, 2003 = 38, 2005 = 57 • HbA1c ≤10% (mean): 2000 = 52, 2001 = 64, 2002 = 70, 2003 = 72, 2005 = 89 (No further statistics provided on these outcomes)	Positive for both outcomes
Kontopantelis (2012) UK Diabetes 6 years	Interrupted time series. 148 practices, 23,920 patients. Quality score = 17	Quality Outcome Framework (QOF) • Direction: positive reward • Form: cash • Magnitude: Set £ value per point • Chance: certain • Target: process • Frequency: some instances incentivised • Immediacy: annually • Schedule: variable	Pre QOF	**Organisational process** HbA1c testing • HbA1c recorded in year (SD): 2000/1 = 71.1 (45.3), 2001/2 = 77.9 (41.5), 2002/3 = 82.8 (37.7), 2003/4 = 89.2 (31.1), 2004/5 = 93.0 (25.5), 2005/6 = 93.7 (24.3), 2006/7 = 93.5 (24.6) **Disease control** HbA1c levels • HbA1c ≤7.4% (SD): 2000/1 = 45.5 (49.8), 2001/2 = 48.4 (50.0), 2002/3 = 50.2 (50.0), 2003/4 = 52.2 (50.0), 2004/5 = 55.6 (49.7), 2005/6 = 56.4 (49.6), 2006/7 = 59.3 (49.1) • HbA1c ≤10% (SD): 2000/1 = 88.5 (31.9), 2001/2 = 90.4 (29.4), 2002/3 = 90.8 (28.9), 2003/4 = 91.8 (27.4), 2004/5 = 92.6 (26.3), 2005/6 = 92.5 (26.3), 2006/7 = 92.7 (26.0)	Positive for both outcomes
LeBlanc (2017) Canada Diabetes 10 years	Longitudinal. 583 physicians, 83,580 adult patients Quality score = 13	New Brunswick P4P Scheme • Direction: positive reward • Form: cash • Magnitude: set $ value per patient • Chance: certain • Target: process • Frequency: all instances incentivised • Immediacy: ongoing • Schedule: fixed	Pre-incentive scheme	**Organisational Process** HbA1c testing • ≤2 HbA1c tests per year: univariate model OR = 1.16 (p<0.0001); 99%CI (1.11 1.20). Multivariate model OR = 1.23 (p<0.0001); 99%CI (1.18, 1.28) **Disease control** HbA1c levels • All patients: univariate model OR = 0.00; 99%CI (-0.03, 0.02). Multivariate model OR = -0.01; 99%CI (-0.03, 0.02 • HbA1C 6.5% to 7.0%: univariate model OR = -0.02 (p<0.0001); 99%CI (-0.04, 0.01). Multivariate model OR = -0.02 (p<0.0001); 99%CI (-0.04, 0.01). • HbA1C 7.1% to 8.9%: univariate model OR = 0.03; 99%CI (-0.01, 0.08). Multivariate model OR = 0.02; 99%CI (-0.02, 0.06). • HbA1C ≥9%: univariate model OR = 0.04; 99%CI (-0.06, 0.15).Multivariate model OR = 0.00; 99%CI (-0.10, 0.10)	Positive organisational process No effect for disease control
Mandel (2007) USA Asthma 26 months	Repeated measures. 44 paediatric practices 13 380 children. Quality score = 16	Cincinnati Children’s Hospital Medical Center asthma improvement collaborative • Direction: positive reward • Form: cash • Magnitude: % of fee schedule • Chance: certain • Target: process • Frequency: some instances incentivised • Immediacy: unclear • Schedule: variable	Pre-incentive scheme	**Organisational process** Asthma action plan ownership. • 19 (70%) achieved the 80% threshold for the PAAP. The cumulative percentage of the network all-payer asthma population receiving “perfect care” increased from 4% to 88%, with 18 of 44 practices (41%) achieving a perfect care percentage of 95% or greater **(**no statistics reported)	Positive
Pape (2015) UK Diabetes 1 year	Before and after study. 1 primary care trust, 6,142 patients. Quality score = 18	Quality Outcome Framework "stretch" scheme (QOF+) • Direction: positive reward • Form: cash • Magnitude: Set £ value per point • Chance: certain • Target: process • Frequency: some instances incentivised • Immediacy: annually • Schedule: variable	Pre QOF+	**Disease control** HbA1c levels HbA1c of ≤8%: • Exception reporting Baseline = 0.085, Secular trend effect = 0.001 (p = 0.910), QOF+ baseline = 0.060 (p = 0.018) • Controlled Patients Baseline = 0.725, Secular trend effect = 0.015 (p = 0.005), QOF+ baseline = 0.002 (p = 0.968) HbA1c of ≤9%: • Exception reporting Baseline = 0.062, Secular trend effect = 0.001 (p = 0.891), QOF+ baseline = 0.043 (p = 0.049) • Controlled Patients Baseline = 0.822, Secular trend effect = 0.015 (p = 0.002), QOF+ baseline = 0.003 (p = 0.934)	No effect
Rosenthal (2005) USA Diabetes 1 year	Quasi-experimental. 205 physician groups, 1,174,294 patients. Quality score = 18	PacifiCare P4P program • Direction: positive reward • Form: cash • Magnitude: set $ value per patient once target met • Chance: certain • Target: process • Frequency: some instances incentivised • Immediacy: quarterly • Schedule: fixed	Same performance figures but no financial incentives	**Organisational process** HbA1c testing Intervention group • Pre Quality Incentive Programme—62.0%, after QIP 64.1%, • Difference (Post-pre), 2.1% (SE 1.0) • P value .02 Control group • Pre Quality Incentive Programme—62.0%, after QIP 64.1%, • Difference (Post-pre), 2.1% (SE 1.0) • P value .02	No effect
Vamos (2011) UK Diabetes 1 year	Interrupted time series. 422 general practices 154 945 patients. Quality score = 15	Quality Outcome Framework (QOF) • Direction: positive reward • Form: cash • Magnitude: Set £ value per point • Chance: certain • Target: process • Frequency: some instances incentivised • Immediacy: annually • Schedule: variable	Pre-QOF	**Organisational process** HbA1c measured • HbA1c measured (95% CI)- 1997, by quintile: 32.8 (31.8–33.7), 31.2 (30.2–32.0), 34.6 (33.7–35.6), 32.2 (31.2–33.0), 37.7 (36.7–38.7) • HbA1c measured (95% CI)- 2005, by quintile: 74.0 (73.4–74.6), 76.4 (75.8–76.9), 77.3 (76.7–77.8), 73.9 (73.3–74.5), 76.2 (75.6–76.8) **Disease control** HbA1c mean levels • HbA1c mean (95% CI)- 1997, by quintile, 7.6 (7.5–7.7), 7.6 (7.5–7.7), 7.7 (7.6–7.8), 7.5 (7.4–7.6), 8.2 (8.1–8.3) • HbA1c mean (95% CI)- 2005, by quintile, 7.5 (7.5–7.5), 7.4 (7.4–7.4), 7.4 (7.4–7.4), 7.5 (7.4–7.5), 7.4 (7.4–7.5) • Baseline proportion of patients meeting HbA1c <7.0% in 1997: 35.3, 95% CI = 31.0–39.7, p<0.05 • Annual change before introduction of P4P: 2.0,95% CI = 1.3–2.7, p<0.05 • Annual change in the year P$P introduced: 0.8, 95% CI = -1.8–3.5, • Annual change after P4P was introduced: -2.2, 95% CI = -4.0- -0.4, p<0.01	No effect
Young (2007) USA Diabetes 2 years	Interrupted time series. 334 Primary care physicians, unknown number of patients. Quality score = 16	Rochester (New York) Individual Practice Association P4P program • Direction: positive reward % avoidance of penalty • Form: cash • Magnitude: % of incentive pool comprised of % of physician fees • Chance: certain • Target: process • Frequency: some instances incentivised • Immediacy: annually • Schedule: variable	Pre-incentive scheme	**Organisational process** HbA1c testing • Adherence rates: mean (SD) pre-intervention: 1999 = 0.56 (0.23), 2000 = 0.57 (0.19), 2001 = 0.59 (0.17) • Adherence rates: mean (SD) post-intervention: 2002 = 0.62 (0.17), 2003 = 0.61 (0.18), 2004 = 0.63 (0.18) • Change in adherence rate: 2000–2001 = 0.018; 2001–2002 = 0.026, p<0.05 • Difference in rate of change (2001–2000) (vs (2002–2004) = 0.009 (no p value given)	No effect

**P4P =** Pay for performance **HbA**_**1**_**C =** glycated haemoglobin

*Financial incentive framework (1) **consists of 9 domains: Direction-** whether the reward is positive gain or avoidance of negative penalty; **Form-**nature of incentive e.g. cash, vouchers etc.**; Magnitude–**value of incentive available to participant; **Certainty-** likelihood of receiving incentive if behaviour changes; **Target-** type of behaviour being targeted; **Frequency-** number of instances of behaviour that are incentivised; **Immediacy-** how soon after the behaviour the incentive is provided; **Schedule-** whether the incentive amount its fixed or variable; **Recipient-** who is in receipt of incentives

The size of the studies, in terms of patients, varied widely from 16 children admitted to hospital with an episode of diabetic ketoacidosis who elected to participate in the scheme [32] to 1,174,294 patients with diabetes whose health insurance company, PacifiCare, trialled a pay-for-performance scheme in their California medical groups and compared results to their medical practices in Oregon and Washington [37]. In these cluster randomised implementation studies, the total number of eligible patients was not always clear. We contacted three authors: two were able to confirm patient numbers [37, 41] and one was not [38]. However, from the number of physicians in this latter study we were able to estimate the number of patients.

The overall weightings [4] which reflect the robustness of the study design (Fig 2), number of participants and quality score are summarised in Table 2.

### Features of the financial incentive schemes

Table 2 describes the characteristics of the studies; the key features of the schemes mapped to the financial incentive framework for documenting financial incentive interventions to change health behaviours [30] are listed in the “Intervention” column. The studies are described below using the domains of this framework.

#### Direction and form

One scheme used avoidance of penalty [32], ten studies used positive rewards [33–37, 39–43] and one study used a mixture of avoidance of penalty and positive rewards [38] as the reward component of the incentive scheme. One study looked at a reimbursement scheme in which the insurance company refunded practices for preventative self-management education costs [30], the remaining eleven schemes were cash incentives paid to the clinicians or practice for achieving targets [33–43].

#### Magnitude and certainty

One study looked at a scheme which paid a financial incentive for each HbA1c test that was completed [33]. One study described a scheme which involved receiving a payment for each performance target met or exceeded [37]. One study described a scheme where physicians were paid a set amount per patient that received two HbA1c tests per year [43]. Eight studies looked at a target achievement scheme where there were pre-set “percentage of patients” targets that physicians had to achieve in order to receive the financial incentive [34–36, 38–42] and ten studies had ‘certain’ incentives if practices successfully achieved targets [33, 35–43]. One scheme had an ‘uncertain’ chance of receiving the financial incentive if they changed their behaviour at the start of the scheme (years 2003–2004) as the payments were only paid to top scoring groups [34]. In the second phase of the scheme (years 2005–2007) this was altered and all groups had a certain chance of receiving a payment if they changed their behaviour. Beck’s 2004 [32] study of children with diabetes in Oklahoma had an uncertain chance of receiving a return on the amount spent on the incentive case management scheme, it depended on whether, and how many times, the participant was rehospitalised.

#### Target and frequency

All schemes [30–43] focussed on “process” behaviours, these are clinician actions that are likely to improve health outcomes. All of the studies included in this review were assessing the impact of financial incentives on clinician behaviour. There were four studies that focussed on a single condition, asthma or diabetes [32, 33, 36, 43]. Three of which reported a positive impact on supported self-management behaviour: Beck 2004 [32] (quasi-experimental, 1 hospital, 16 children, D&B = 15); LeBlanc 2017 [43] (longitudinal, 583 physicians, 83,580 adult patients, D&B = 13) and Mandel 2007 [36] (repeated measures, 44 practices, 13,380 children, D&B = 16). The final study, Chien 2012 [33] (quasi-experimental, 118 practices, 5,557 participants, D&B = 13), showed no effect. The rest of the studies looked at multiple condition schemes which included diabetes and reported a mixture of positive results [39, 40], no effect [35, 37, 38, 41, 42] and a negative outcome was reported by Conrad 2013 [34] (quasi-experimental, 19 medical groups, 21, 365 patients, D&B = 10). Two schemes [32, 33] incentivised all instances of the behaviour and the remaining studies had some instances incentivised as they had to reach percentage targets [34–42].

#### Immediacy and schedule

The financial incentive framework [30] defines immediacy as how soon the recipient receives the incentive payment after the behaviour. If the time between behaviour and reward is too long, recipients may not link the two and the incentive will fail to be effective. Eight of the included schemes paid incentives on an annual basis [33–35, 38–42]. Two studies reported an explicit link between performance and payment; Rosenthal 2005 [37] described a scheme which paid a quarterly bonus of $0.23 per member per month for each performance target that was met or exceeded by the physician group and Chien 2012 [33] reporting practices receiving $100 for each patient for which missing care processes were completed. It was unclear in the article by LeBlanc (2017) [43] when the physicians received the payment for achieving the target of two HbA1c tests per year.

In the only asthma study included [36], the Cincinnati asthma improvement collaborative comprised of three stages with two different payment phases: all awards were assessed on 31 December 2004 and first-level fee schedule increases implemented from 1 May 2004 through to 31 December 2005; second and third-level fee schedule increases effective from 1 March 2005, through to 31 December 2005.

Beck 2003 [32] developed a 15-month scheme with a less tangible reward of reduced healthcare costs where they calculated financial impact of participation in the programme versus the healthcare costs per participant and non-participant.

#### Recipients

Although all studies looked at a financial incentive paid to either the clinician or the practice, the papers differed in the way in which they reported numbers of study participants: nine articles noted number of patients [32–35, 39, 40, 42, 43]; seven referred to the number of practices/medical groups [33–37, 39, 40, 42, 43]; one study discussed a primary care trust (administrative body responsible for primary healthcare services in England) [41] and one discussed number of physicians [38]. Fagan [35] described an intervention for individuals with diabetes aged 65 years plus, two studies focussed on a targeted population of children [32, 36] and Chien 2012 [33] evaluated the impact of a scheme where lower socio-economic populations were targeted.

### Impact of the schemes on process, behavioural and health outcomes

**Fig 3:**
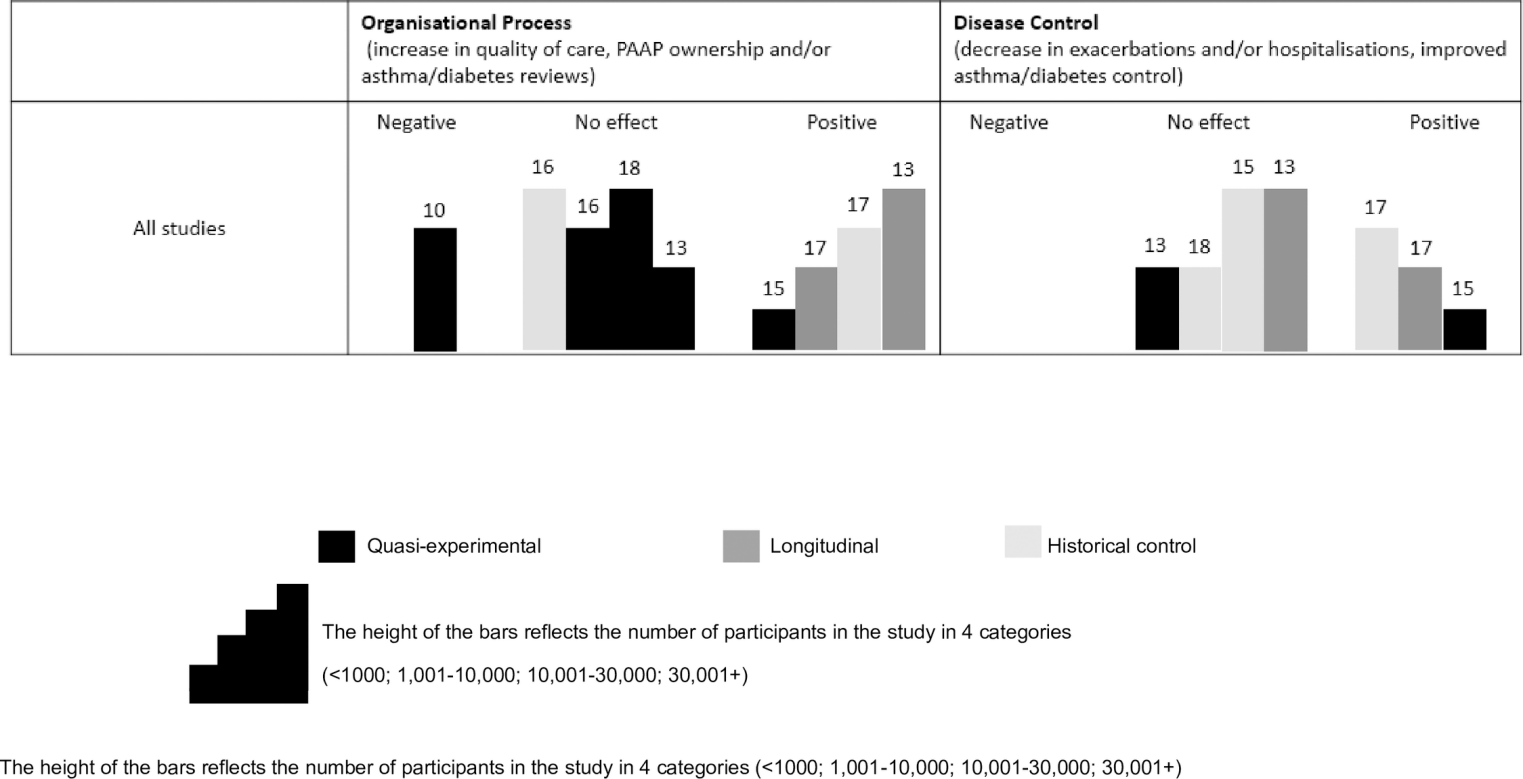
Harvest plot. Illustrating the impact of financial incentives schemes on organisational process and disease control outcomes. Notes: Each bar represents an individual study. The colour of the bar indicates the study design, the height of the bar reflect the number of participants in the study and the number is the Downs and Black quality score. The decisions that underpin this plot are detailed in S1 Table.

Table 2 summarizes the key findings from each of the studies and Fig 3 illustrates our synthesis with supporting information in S1 Table.

#### Organisational process

One study described an asthma improvement collaborative in Cincinnati [36] (repeated measures, 44 practices, 13,380 children, D&B = 16) which consists of a three level reward system. Practices had to reach a set target in each level in order to be eligible to proceed to the next level. Written action plans for patients with asthma were part of the criteria for the third level of the reward system. Authors concluded that the asthma pay-for-performance scheme had a positive impact on the proportion of patients with asthma receiving “perfect care” which increased from 4% before the intervention to 88% after. “Perfect care” was assessed on performance of components including: provision of a written action plan; provision of controller medication (if required); and recording patients’ control based on National Heart, Lung, and Blood Institute guideline recommended classification.

Nine studies reported proportion of patients who received HbA1c tests [33–35, 37–40, 42, 43]. Six reported that the financial incentive scheme had no effect [33, 35, 37, 38, 42], three reported that financial incentives had a positive effect on increasing frequency of HbA1c testing [39, 40, 43] and one study reported negative impact on the number of HbA1C tests performed [34]. Fagan 2010 [35] (Quasi-experimental, 20,943 65+ year old patients, D&B = 16) found that although the intervention group improved, it did not improve as much as the comparison group; the authors concluded that the study did not generate significant evidence to support a pay for performance scheme.

Chien [33] (quasi-experimental, 118 practices, 5,557 participants, D&B = 13) found no statistically significant improvement in patterns of care or clinical outcomes. They identified that younger adults and those with more comorbidities were less likely to receive recommended care and experienced a diabetes-related emergency department visit more often. However, two studies noted that practices in lower socio-economic status areas require additional support to overcome barriers [37, 39].

#### Disease control

Six studies reported on test results for HbA1c levels [33, 39–43], with four studies reporting no effect [33, 41, 42] and two reporting a positive effect [39, 40]. One study evaluated an intensive case management scheme offered to 16 children who had been hospitalised after an incident of diabetic ketoacidosis [32]. They reported that participation in the intensive program was associated with fewer hospitalisations resulting in lower costs for participants ($1063 per individual) than non-participants ($2396 per individual).

#### Individual behavior

None of the studies reported on self-efficacy, activation or adherence to medication which we had classified as “individual behaviour”.

## Discussion

### Statement of principal findings

A total of 12 papers (three diabetes; one asthma; eight multiple condition schemes including diabetes but not asthma) reporting on self-management interventions met the inclusion criteria and were included in the review. The impact of financial incentives paid to healthcare professionals for implementing self-management to patients with asthma or diabetes is inconsistent. Although most showed no effect [33, 35, 37, 38, 41, 42, 43] or a positive impact [32, 36, 39, 40, 43] on organisational process or disease control outcomes, one study targeting organisational processes showed a negative effect on the proportion of people with HbA1c testing [34]. We found no articles which analysed the impact of financial incentives on individual behaviour outcomes.

### Interpretation of findings in relation to previously published work

The schemes targeted a range of organisational and health outcomes including: programme participation, asthma action plan ownership, HbA1c testing, HbA1C level testing and hospitalisations. However, we did not find any studies matching our inclusion criteria which looked at individual behavioural outcomes, identifying a gap in the research literature. For the organisational process outcomes five of the studies reported positive results [32, 36, 39, 40, 43], five reported no statistically significant effect [33, 35, 37, 38, 42] and one reported that the financial incentive scheme had a negative impact on results [34]. In the studies targeting disease control outcomes; three reported positive effects [32, 39, 40] and four reported no effect [33, 41, 42, 43]. Effective implementation strategies involve a multifaceted approach accommodating patient, professional and organisational aspects [5] but financial incentive schemes do not incorporate all of these aspects. Typically, they focus on the professionals, the organisations, or the patients separately but do not take a whole systems approach which proposes that all of these aspects require inclusion for successful implementation.

The financial incentives schemes were diverse and incorporated features across all the domains of the financial incentives framework (1). It was difficult to draw conclusions on which type of scheme was the most effective in changing healthcare professionals’ behaviour in relation to providing supported self-management to individuals with asthma or diabetes. Four of the studies that reported no statistically significant effect noted that the magnitude of the financial incentive might have contributed to the lack of effect [33–35, 37]. If the health care professional deems the size of the incentive potentially too modest for the effort and money spent on achieving the target, they are unlikely to change their behaviour.

The one negative result is a reminder that providing financial incentives may have unintended consequences and the implementation of financial incentive schemes must be approached with caution. Previous work has identified the potential negative impact of financial incentives schemes and produced a checklist to prevent inappropriate implementation [45]. Glazsiou’s checklist consists of nine questions and is divided into two parts “Part A: Is a financial incentive appropriate?” and “Part B: Implementation”. All six questions in Part A must be answered yes before continuing to considering implementation in Part B. One question in the checklist addresses the potential for unintended consequences and specifically highlights harm to the patient-clinician relationship. They provide evidence from a report showing that some UK clinicians became reluctant to register patients with complex poorly controlled conditions that would make it difficult for them to achieve their QOF targets [46]. Within QOF guidelines, practices are able to exclude patients from their reporting if the intervention was considered inappropriate, or was declined by the patient. Two studies identified overuse of ‘exception reporting’ as a strategy for potentially achieving more favourable results [39, 41]. Gulliford 2007 [39] raised concerns that an increase in ‘excepted’ cases was a potential reason for high QOF achievements. Pape [41] found that with the introduction of QOF+ (a UK scheme with more ambitious targets than the national QOF scheme), ‘exception reporting’ increased significantly in the indicators for HbA1c and concluded that financial incentives schemes had no significant effect.

When applying the financial incentives framework [30] to schemes for clinicians, the ‘Recipient’ domain does not take into consideration the patient population and whether the scheme was targeted at a specific population, for example lower socio-economic status was a population identified by two authors as having barriers which require additional support [37, 39]. Glazsiou’s checklist for implementing a financial incentive scheme identifies the importance of understanding and assessing potential barriers to changing clinician behaviours [45]. Evidence has shown that while financial incentives have the potential to reduce the inequalities in achievement related to area deprivation, differences do still exist [47], these must therefore be considered when designing future financial incentives schemes aimed at clinician behaviour.

The number of conditions in the scheme, a domain not included in the financial incentives framework [30], was not consistently associated with positive or negative findings and further research is required into whether an incentive scheme focussing on a single condition rather than multiple conditions would produce more positive results.

### Strengths and limitations

The heterogeneity of methodologies used in studies investigating financial incentives paid to health care professionals for providing self-management education to their patients with asthma or diabetes made it difficult to compare studies. Therefore, we adopted the approach of Pinnock 2015 [4] and classified papers by methodology robustness, number of participants and quality score. A number of questions on the quality checklist employed in this review [26] were not appropriate for the papers included which led to low quality scores. A recently published quality checklist for reporting implementation studies may provide a framework better able to assess the quality of implementation research [29].

All studies were conducted in either the United States of America, Canada or the UK which limits the generalisability of the findings. Research looking at financial incentives aimed at healthcare professionals uses self-reporting data which presumes that all information provided is accurate and truthful.

All the studies were nonrandomised studies which are inherently more biased than randomised control trials [48], though the risk of bias in the included studies was assessed as low or unclear in the majority of the studies. Selection bias, purposive sampling, and selective outcome reporting were also identified in the selected studies.

We were unable to complete funnel plots to measure the extent of publication bias as we conducted a narrative analysis not a meta-analysis due to the heterogeneity of the study designs. However, the results of the included studies were a mixture of positive, no effect and negative on health and process outcomes in self-management of asthma or diabetes which suggests that there was not a high percentage of publication bias [49].

Time and resource constraints meant that the initial screening of title and abstracts was conducted by a single reviewer. However, training and quality assessment were undertaken on 5% of title/abstracts screened to reduce subjectivity and minimise potential inaccuracies. Full text screening and data extraction was completed by two reviewers.

### Implications for future research, clinical care and policy

The limited number of studies investigating the impact of financial incentives on the implementation of asthma or diabetes self-management identifies a gap in the literature where further research is required. In particular, we only identified one study investigating the impact of financial incentives on the implementation of asthma self-management [36]. There is a further gap in research assessing the impact of financial incentives paid to healthcare professionals, on behavioural outcomes such as self-efficacy, activation or adherence to medication; no studies were identified in this area.

Further research is needed to confirm findings and understand the process by which financial incentives impact (or not) on care. Determinants of how financial incentives impact on organisation of care and health outcomes are multifactorial and complex. Results from this systematic review show that as well as money, there are other factors influencing healthcare professionals’ behaviour in delivering self-management for asthma and diabetes. Smaller practices may lack the infrastructure that is required to improve quality of care [38] and practices with a patient population of low socioeconomic status face barriers that make financial incentives schemes less effective in these areas [37, 39]. The use of ‘exception reporting’ for individuals who do not meet QOF (or other financial incentives scheme’s) guidelines needs to be monitored to ensure that individuals who require specialised, complex or more critical care are not being overlooked. When producing incentive schemes designers need to consider: the existing infrastructure in the organisation; target populations; the size of the incentive and time; effort and resources required to implement change; as well as unintended consequences.

## Conclusion

The evidence provided in this systematic review showed mixed results for whether financial incentives have an impact on behaviour change in healthcare professionals to provide self-management to individuals with asthma or diabetes. Due to the diversity of the schemes, it is difficult to draw conclusions on what aspects of the incentives are most effective. However, size of financial incentives, exception reporting and socio-economic status of patient population were all reported as being influential. The number of conditions in an incentive scheme, i.e. targeted on one condition or multiple condition scheme, was not associated with the success of the scheme. Further research is required in order to understand the complex nature of behaviour changing interventions on healthcare professionals in relation to increasing self-management in individuals with asthma or diabetes.

## Supporting information

S1 Appendix Published protocol. (PDF)

S2 Appendix Search strategy for identification of studies for financial incentives in relation to self-management of asthma or diabetes. (PDF)

S1 Table Supporting information for Harvest plots. (PDF)

S2 Table PRISMA (Preferred Reporting Items for Systematic Reviews and Meta-Analyses) checklist. (PDF)

S1 Fig Risk of bias summary. (TIF)
