# Supplementary material for: The impact of financial incentives on the implementation of asthma or diabetes self-management: A systematic review
Source: PLoS One. 2017 Nov 6;12(11):e0187478. doi: 10.1371/journal.pone.0187478 (PMC5673190; doi:10.1371/journal.pone.0187478)
Supplement: S1 Appendix — (PDF) [file pone.0187478.s001.pdf]

The impact of incentives on the implementation of asthma or diabetes self-management

Protocol for a systematic review

Research Team

Tracy Jackson	Asthma UK Centre for Applied Research, Usher Institute of Population Health Sciences and Informatics, The University of Edinburgh
Professor Liam Heaney	Asthma UK Centre for Applied Research, Centre for Infection & Immunity, Queen's University Belfast
Professor Mike Shields	Asthma UK Centre for Applied Research, Centre for Infection & Immunity, Queen's University Belfast
Io Hui Chi Yan	Asthma UK Centre for Applied Research, Usher Institute of Population Health Sciences and Informatics, The University of Edinburgh
Dr Hilary Pinnock	Asthma UK Centre for Applied Research, Usher Institute of Population Health Sciences and Informatics, The University of Edinburgh

Library Services

Marshall Dozier	College of Medicine and Veterinary Medicine, University of Edinburgh

Funding This work is funded by The University of Edinburgh CMVM PhD Studentship [Asthma UK Centre for Applied Research PHD/14/16]

THE UNIVERSITY of EDINBURGH Queen's University Belfast Asthma UK Centre for Applied Research

Table of Contents

Introduction

Aim of the systematic review

Research questions

Plan of investigation

Identification of studies
Search strategy
Study selection
Exclusion criteria
Data extraction
Quality appraisal and weighting
Analysis and synthesis

Conflicts of interest

Dissemination

Timetable

References

Appendix I: Search strategy

Introduction

Asthma and diabetes in the United Kingdom

Asthma affects 5.4 million people in the United Kingdom (UK)[1] and each day three people die because of asthma[2]. Supported self-management including education and Personalised Asthma Action Plans (PAAPs) have consistently been proven to improve asthma control, minimise exacerbations and reduce emergency use of healthcare resources[3-7]. The British Guideline on the Management of Asthma recommends that all individuals with asthma should be provided with self-management education and offered a PAAP[8]. However, as identified by Asthma UK, ownership of PAAPs remains low with only 24% of individuals with asthma in the UK being in possession of a PAAP[9].

There are 3.9 million people in the UK who have been diagnosed with diabetes and it is estimated that approximately 590,000 are as yet undiagnosed[10]. Self-management of diabetes, including lifestyle changes, adherence to medication and monitoring and adjusting dosages accordingly can greatly improve quality of life. However, Diabetes UK have found that 42% of individuals with Type 2 diabetes do not feel confident managing their diabetes and in England and Wales, only 16% of individuals with diabetes were offered an education course when first diagnosed[11]. Within the UK there are eight annual checks that individuals with diabetes should receive to manage their diabetes effectively and reduce the possibility of complications. Only 36% of individuals with diabetes are meeting the targets set for these annual checks[12], though this has increased since the National Diabetes Audit 2011–1213 which reported that the percentage of individuals meeting the targets was 21%.

Quality and Outcome Framework (QOF) for Long Term Conditions (LTC)

While it has been routine in the past for doctors to have their quality of care reviewed, in recent years this reviewing has been conducted by external bodies and financial incentives have been introduced to achieve set targets hoping to improve "good practice" in clinical care. Since 2004 in the UK, clinical performance targets are included in the contracts of General Practitioners (GPs), enabling them to gain additional income through financial incentives received from attaining targets within the QOF[14]. The QOF focuses on nineteen clinical areas including asthma and diabetes[15]. The QOF targets for asthma are: establishing and maintaining an asthma register and providing an annual review to assess asthma control, respond to assessment and adjustment of management and explore perceptions and support self-management[16], however self-management is not an incentivised target. In contrast, QOF targets for treating individuals with diabetes include referring newly diagnosed individuals with diabetes to a structured education programme within nine months of being added to the diabetes register[17].

Northern Ireland's Directed Enhanced Service (DES) for asthma

Established in 2008, Northern Ireland's (NI) DES includes a scheme which pays a financial incentive, in addition to QOF, to general practices that provide self-management education, including a PAAP to people with asthma[18]. There are three levels to the financial incentive depending on whether the general practice provides self-management education to 50%, 65% or 75% of the individuals with

asthma on their practice register[19]. Asthma UK (2013), estimated that the proportion of people with asthma who own a PAAP in NI was 60%, which is double the proportion in Scotland, and identified the DES as the major contributor to this.

Our overall programme of work

This systematic review is part of a research project that will investigate the increase in PAAP ownership in NI, identify what actions practices implemented in order to achieve this improvement and measure the effectiveness of the DES. While the research project is focussed on asthma, literature on diabetes has been included in this systematic review due to diabetes being a LTC comparative condition with existing incentive schemes. By reviewing the literature on the impact of financial incentives used to implement asthma self-management and diabetes checks, this review will provide the underpinning evidence for this research project. As part of a wider programme of work within the Asthma UK Centre for Applied Research investigating the implementation of supported self-management this project will directly inform programme 1 of the AUKCAR; "How do we empower and enable people to take control of their asthma so they can live full and active lives?". Implementation of supported self-management is challenging[17], and this work will be of interest to policy makers and commissioners and providers of healthcare services seeking to embed self-management into routine clinical care.

Aim of the systematic review

To systematically review the evidence investigating the impact of financial incentives on organisational process outcomes, individual behavioural outcomes, and health outcomes for individuals with asthma or diabetes.

Research questions

- What is the impact of financial incentives for implementation of asthma or diabetes supported self-management on professional/organisational process outcomes (ownership of PAAPs, asthma/diabetes reviews)
- What is the impact of financial incentives for implementation of asthma or diabetes supported self-management on disease control (asthma/diabetes control, risk of exacerbation, hospital admittance rates)
- What is the impact of financial incentives for implementation of asthma or diabetes supported self-management on behaviour of individuals with asthma or diabetes (self-efficacy, activation, adherence to preventer medication, adherence to insulin medication)

Outcome measures

We are interested in primary and secondary outcomes in relation to the 3 research questions, these are detailed in table 1.

Table 1: Primary and secondary outcomes

	Primary	Secondary
1. Organisational process	• asthma - ownership of a personalised asthma action plan<br>• diabetes - attendance at a patient training/self-management course	• attendance at reviews supporting self-management
2. Measure of disease control	• asthma - symptom control<br>• asthma - exacerbations<br>• diabetes - glycaemic control	• unscheduled care<br>• diabetes – blood pressure control
3. Individual behaviour	• self-efficacy	• activation<br>• adherence to medication

Plan of investigation

We will follow the procedures described in the Cochrane Handbook for Systematic Reviews of Interventions**.**

Identifications of studies

A PICOS search strategy, shown in table 2, will be utilised to search databases, examine bibliographies and identify unpublished and in progress studies.

Search strategy

- Databases searched: Cochrane Central Register of Controlled Trials (CENTRAL); Cochrane Database of Systematic Reviews (CDSR); MEDLINE; PsychInfo; CINAHL; ScienceDirect; Web of Science; Embase
- References from published studies: The bibliographies of all eligible studies will be examined to identify potential studies for inclusion.
- Unpublished and in progress studies: UK Clinical Research Network: Portfolio Database and the metaRegister of Controlled Trials will be included in the review.
- It is anticipated that most of the studies retrieved will be reporting on implementation studies, therefore a broad range of studies have been included in the search strategy.

Table 2: PICOS search strategy

Population	• Healthcare professionals incentivised (or whose organisation is incentivised) to provide self-management<br>• Individuals with asthma or diabetes receiving care from an organisation which is receiving financial incentivise
Intervention	• Any financial incentive provided to a healthcare organisation and/or healthcare professionals that is designed to improve supported self-management in asthma or diabetes
Comparison	• Healthcare professionals not incentivised (or whose organisation is not incentivised) to provide self-management.<br>• Individuals with asthma or diabetes who are receiving usual, non-incentivised care
Outcomes	• Organisational process: increase in quality of care, PAAP ownership and/or asthma/diabetes reviews<br>• Disease control: decrease in exacerbations and/or hospitalisations, improved asthma/diabetes control<br>• Individual behaviour: self-efficacy, activation, adherence to medication
Setting	• Any healthcare setting
Study Design	• Randomised controlled trials (RCTs)<br>• Quasi -RCTs<br>• Controlled before and after studies<br>• Interrupted time series<br>• Repeated measures

Study selection

One reviewer (TJ) will conduct the search and download all search results into Endnote.

Training: Two reviewers (TJ and HP) will screen a random selection of 100 papers, compare and discuss decisions in order to reach agreement. This process will be repeated until the reviewers are in agreement with the search criteria and its application to the studies.

Title and abstract screening: One reviewer (TJ) will consider the remaining titles and abstracts rating

them “full text screening required” or “reject”. Full text will then be retrieved for the potentially relevant papers.

Full text screening: Two reviewers (TJ and IH) will independently review the full text papers, with a discussion between two reviewers to resolve disagreements with a third reviewer (HP) being involved when an agreement cannot be achieved.

Exclusion criteria

- Reviews, systematic reviews, and meta-analyses
- Guidelines
- Study protocols (though we will search for published results if we identify a relevant protocol)
- Surveys
- Editorials, and opinion pieces
- Abstracts (though we will search for published results if we identify a relevant abstract)
- Letters, case reports, audits,,
- Articles where incentive recipient is the patient
- Articles where the incentive is not financial
- Articles where the focus is not on supported self-management

Data extraction

Data will be extracted from included papers by one reviewer and checked by a second using a customised data extraction tool which will be piloted prior to the review to ensure it captures all relevant information and is interpreted consistently. Discussion between two reviewers will resolve disagreements with a third reviewer being involved when an agreement cannot be achieved. We will extract details about the interventions under the following headings: “setting”, “financial incentive”, “methodology” and “outcomes”.

Forward citations of the included studies will be checked for descriptions of interventions, nested qualitative studies, and process evaluations in order to provide context. If the descriptions in the papers are inadequate, authors will be contacted and a short qualitative interview may be undertaken in order to provide further information on the intervention.

Quality appraisal and weighting

Randomised controlled trials papers selected for retrieval will be assessed for quality by one reviewer and checked by a second using methods detailed in section six of the Cochrane Handbook for Systematic Reviews of Interventions. Seven domain-based parameters will be used to assess quality; adequate sequence generation; allocation concealment; blinding of participants and personnel; blinding of outcome assessment; incomplete outcome data addressed; selective reporting and free of other bias. Parameters will be graded as: A- low risk of bias; B- moderate risk of bias; C – high risk of bias and an overall assessment for each controlled trial using the same three criteria will be made. For non-randomised interventions studies, the Cochrane Effectiveness and Practice Organisation of Care

(EPOC) guidelines will be used for assessment and the Good practice data extraction form used[20.]

A broad range of study design is anticipated as the studies retrieved will be reporting on implementation studies. In order to manage the diverse range of methodologies in these papers, we will weight the included papers. The approach of Pinnock et al (2015) will be adopted and papers will be classified by robustness of methodology, number of participants and the quality score. Methodological quality assessment will be used to assess the agreement of reviewers and any disagreements will be resolved by discussions. In the event that an agreement cannot be reached, a third reviewer will be brought in to mediate.

Analysis and synthesis

Preliminary literature searches have suggested that a limited number of eligible trials with substantial heterogeneity will be identified so meta-analysis will not be appropriate. Therefore, a narrative synthesis will be undertaken. However, if sufficient trials suitable for inclusion in a meta-analysis are identified then the standard procedures described in the Cochrane handbook will be followed.

Asthma and diabetes will initially be analysed separately. We will classify components of the interventions (e.g. whether the financial incentive is paid to the individual (self-employed) healthcare professional or an organisation interventions; payment for process standards (e.g. attendance at a diabetes course) or health outcomes (reduced unscheduled care) We will develop a matrix of interventions shown to be effective or ineffective under the headings of: “organisational process”; “measure of disease control” and “individual behaviour”.

We will then undertake an over-arching synthesis, looking for similarities and differences between the effective and ineffective strategies in the findings of asthma and diabetes studies.

Conflicts of interest

The authors declare that there are no conflicts of interest.

Dissemination

The findings in this study will be presented at conferences, submitted to peer-reviewed journals and is aligned to Programme 1 of the AUKCAR which is “How do we empower and enable people to take controls of their asthma so they can live full and active lives?” This review will also contribute towards the submission of a Population Health Sciences PhD.

Timetable

Months 1 - 6	• Write protocol<br>• Develop search strategy<br>• Search databases<br>• Collect data<br>• Initial data analysis
Months 6-9	• Select papers for review
Month 10	• Prospero registration
Month 11	• Extract data<br>• Quality appraisal
Months 12-15	• Final data analysis<br>• Compose report<br>• Write paper

References

1. Asthma UK. Asthma Facts and FAQs. Asthma UK. 2014. Available from http://www.asthma.org.uk (accessed February 2015)
2. Royal College of Physicians. Why asthma still kills, The National Review of Asthma Deaths (NRAD). Royal College of Physicians. 2014. Available from http://www.rcplondon.ac.uk (accessed February 2015)
3. Taylor SJC, Pinnock H, Epiphaniou E, Pearce G, Parke H. A rapid synthesis of the evidence on interventions supporting self-management for people with long-term conditions. (PRISMS Practical Systematic Review of Self-Management Support for long-term conditions) Health Serv Deliv Res 2014; 2:54
4. Gibson PG, et al. Self-management education and regular practitioner review for adults with asthma. Cochrane Database of Systematic Reviews 2002, Issue 3. Art.No:CD001117
5. Tapp S, et al. Education interventions for adults who attend the emergency room for acute asthma. Cochrane Database of Systematic Reviews 2007, Issue 3. Art. No.: CD003000
6. Gibson PG, et al. Written action plans for asthma: an evidence-based review of the key components. Thorax 2004;59:94–99
7. Powell H, et al. Options for self-management education for adults with asthma. Cochrane Database of Systematic Reviews 2002, Issue 3. Art. No.: CD004107
8. British Thoracic Society, Scottish Intercollegiate Guideline Network, A national clinical guideline: The British Guideline on the Management of Asthma. British Thoracic Society. 2014. Available from http://www.sign.ac.uk (accessed April 2015)
9. Asthma UK. Compare your care report. Asthma UK. 2013 Available from http://www.asthma.org.uk (accessed February 2015)
10. Diabetes UK. Diabetes Facts and Stats. Diabetes UK. 2015. Available from www.diabetes.org.uk (Accessed October 2015)
11. Diabetes UK. 42% of people with Type 2 diabetes “not confident managing their condition”. Diabetes UK. 2015. Available from www.diabetes.org.uk (Accessed October 2015)
12. HSCIC: National Diabetes Audit 2012/13: Report 1: Care Processes and Treatment Targets. Available from http://www.hscic.gov.uk (accessed October 2015)
13. HSCIC: National Diabetes Audit 2011/12: Report 1: Care Processes and Treatment Targets. Available from http://www.hscic.gov.uk (accessed October 2015)
14. Downing A. et al. Do the UK government's new Quality and Outcomes Framework (QOF) scores adequately measure primary care performance? A cross-sectional survey of routine healthcare data. *BMC Health Services Research* 2007; 7:166
15. NHS Employers. 2014/15 General Medical Services (GMS) Contract quality and Outcomes Framework (QOF). NHS Employers. 2014. Available from http://www.nhsemployers.org (accessed October 2015)
16. Pinnock H, Fletcher M, Holmes S, Keeley D, Leyshon J, Price D, Russell R, Versnel J, Wagstaff B. Setting the standard for routine asthma consultations: a discussion of the aims, process and outcomes of reviewing people with asthma in primary care. Prim Care Respir J 2010; 19: 75-83

17. NHS Employers. 2015/16 General Medical Services (GMS) Contract quality and Outcomes Framework (QOF). NHS Employers. 2015. Available from http://www.nhsemployers.org (accessed October 2015)
18. Department of Health, Social Services, and Public Safety. The Primary Medical Services (Directed Enhanced Services) Directions (Northern Ireland) 2008. Available from http://www.dhsspsni.gov.uk (accessed February 2015)
19. Pinnock H, Epiphaniou E, Pearce G, Parke HL, Greenhalgh T, Sheikh A, Griffiths CJ, Taylor SJC. Implementing supported self-management for asthma: a systematic review of implementation studies. *BMC Medicine* 2015; 13:127
20. Effective Practice and Organisation of Care (EPOC). EPOC Resources for review authors. Oslo: Norwegian Knowledge Centre for the Health Services; 2015. Available at: http://epoc.cochrane.org/epoc-specific-resources-review-authors

Appendix I: Search strategy

Search terms for Cochrane Central Register of Controlled Trials (CENTRAL); Cochrane Database of Systematic Reviews (CDSR); CINAHL; ScienceDirect; Web of Science

(asthma* or diabet*)

AND

({managed care program} OR {fee for service} OR {fee-for-service} OR {reimbursement} OR {financial incentiv*} OR {pay for performance} OR {pay-for-performance} OR {cash transfer*} OR {incentive reimbursement*} OR {direct* enhance* service})

AND

({self management} OR {self-management} OR {self-care} OR {self care} OR {asthma action plan})

Search terms for MEDLINE

1. exp Asthma/
2. exp Diabetes Mellitus, Type 1/ or exp Diabetes Mellitus, Type 2/ or exp Diabetes Mellitus/
3. 1 or 2
4. exp Managed Care Programs/
5. exp Reimbursement, Incentive/
6. ("financial incentiv*" or "pay for performance" or "pay-for-performance" or "cash transfer*" or "incentive reimbursement*" or "directed enhanced service").mp. [mp=title, abstract, original title, name of substance word, subject heading word, keyword heading word, protocol supplementary concept word, rare disease supplementary concept word, unique identifier]
7. 4 or 5 or 6
8. exp self care/ or exp blood glucose self-monitoring/ or exp self administration/
9. ("self management" or "self-management" or "management" or "self-care" or "self care" or "asthma action plan").mp. [mp=title, abstract, original title, name of substance word, subject heading word, keyword heading word, protocol supplementary concept word, rare disease supplementary concept word, unique identifier]
10. 8 or 9
11. 3 and 7 and 10

Search terms for PsychInfo

1. exp Asthma/
2. exp Diabetes/ or exp Diabetes Mellitus/

3. 1 or 2
4. exp Fee for Service/
5. exp Incentives/ or exp Monetary Incentives/
6. exp Managed Care/
7. ("financial incentiv*" or "pay for performance" or "pay-for-performance" or "cash transfer*" or "incentive reimbursement*" or "directed enhanced service").mp. [mp=title, abstract, heading word, table of contents, key concepts, original title, tests & measures]
8. 4 or 5 or 6 or 7
9. exp Self Care Skills/ or exp Self Management/
10. exp Self Monitoring/ or exp Self Management/
11. ("self management" or "self-management" or "management" or "self-care" or "self care" or "asthma action plan").mp. [mp=title, abstract, heading word, table of contents, key concepts, original title, tests & measures]
12. 9 or 10 or 11
13. 3 and 8 and 12

Search terms for Embase

1. exp asthma/
2. exp diabetes mellitus/
3. 1 or 2
4. reimbursement, incentive.mp. or exp reimbursement/
5. exp medical fee/
6. exp managed care/
7. ("financial incentiv*" or "pay for performance" or "pay-for-performance" or "cash transfer*" or "incentive reimbursement*" or "directed enhanced service").mp. [mp=title, abstract, heading word, drug trade name, original title, device manufacturer, drug manufacturer, device trade name, keyword]
8. 4 or 5 or 6 or 7
9. exp self care/
10. ("self management" or "self-management" or "management" or "self-care" or "self care" or "asthma action plan").mp. [mp=title, abstract, heading word, drug trade name, original title, device manufacturer, drug manufacturer, device trade name, keyword]
11. 9 or 10
12. 3 and 8 and 11
