# Supplementary material for: The impact of financial incentives on the implementation of asthma or diabetes self-management: A systematic review
Source: PLoS One. 2017 Nov 6;12(11):e0187478. doi: 10.1371/journal.pone.0187478 (PMC5673190; doi:10.1371/journal.pone.0187478)
Supplement: S2 Appendix — (PDF) [file pone.0187478.s002.pdf]

Search terms for Cochrane Central Register of Controlled Trials (CENTRAL); Cochrane Database of Systematic Reviews (CDSR); CINAHL; ScienceDirect; Web of Science

(asthma* or diabet*)

AND

({managed care program} OR {fee for service} OR {fee-for-service} OR {reimbursement} OR {financial incentiv*} OR {pay for performance} OR {pay-for-performance} OR {cash transfer*} OR {incentive reimbursement*} OR {direct* enhance* service})

AND

({self management} OR {self-management} OR {self-care} OR {self care} OR {asthma action plan})

Search terms for MEDLINE

1. exp Asthma/
2. exp Diabetes Mellitus, Type 1/ or exp Diabetes Mellitus, Type 2/ or exp Diabetes Mellitus/
3. 1 or 2
4. exp Managed Care Programs/
5. exp Reimbursement, Incentive/
6. ("financial incentiv*" or "pay for performance" or "pay-for-performance" or "cash transfer*" or "incentive reimbursement*" or "directed enhanced service").mp. [mp=title, abstract, original title, name of substance word, subject heading word, keyword heading word, protocol supplementary concept word, rare disease supplementary concept word, unique identifier]
7. 4 or 5 or 6
8. exp self care/ or exp blood glucose self-monitoring/ or exp self administration/

9. ("self management" or "self-management" or "management" or "self-care" or "self care" or "asthma action plan").mp. [mp=title, abstract, original title, name of substance word, subject heading word, keyword heading word, protocol supplementary concept word, rare disease supplementary concept word, unique identifier]

10. 8 or 9

11. 3 and 7 and 10

Search terms for PsychInfo

1. exp Asthma/

2. exp Diabetes/ or exp Diabetes Mellitus/

3. 1 or 2

4. exp Fee for Service/

5. exp Incentives/ or exp Monetary Incentives/

6. exp Managed Care/

7. ("financial incentiv*" or "pay for performance" or "pay-for-performance" or "cash transfer*" or "incentive reimbursement*" or "directed enhanced service").mp. [mp=title, abstract, heading word, table of contents, key concepts, original title, tests & measures]

8. 4 or 5 or 6 or 7

9. exp Self Care Skills/ or exp Self Management/

10. exp Self Monitoring/ or exp Self Management/

11. ("self management" or "self-management" or "management" or "self-care" or "self care" or "asthma action plan").mp. [mp=title, abstract, heading word, table of contents, key concepts, original title, tests & measures]

12. 9 or 10 or 11

13. 3 and 8 and 12

Search terms for Embase

1. exp asthma/

2. exp diabetes mellitus/

3. 1 or 2

4. reimbursement, incentive.mp. or exp reimbursement/

5. exp medical fee/

6. exp managed care/

7. ("financial incentiv*" or "pay for performance" or "pay-for-performance" or "cash transfer*" or "incentive reimbursement*" or "directed enhanced service").mp. [mp=title, abstract, heading word, drug trade name, original title, device manufacturer, drug manufacturer, device trade name, keyword]

8. 4 or 5 or 6 or 7

9. exp self care/

10. ("self management" or "self-management" or "management" or "self-care" or "self care" or "asthma action plan").mp. [mp=title, abstract, heading word, drug trade name, original title, device manufacturer, drug manufacturer, device trade name, keyword]

11. 9 or 10

12. 3 and 8 and 11
