# Supplementary material for: The impact of financial incentives on the implementation of asthma or diabetes self-management: A systematic review
Source: PLoS One. 2017 Nov 6;12(11):e0187478. doi: 10.1371/journal.pone.0187478 (PMC5673190; doi:10.1371/journal.pone.0187478)
Supplement: S1 Table — (PDF) [file pone.0187478.s003.pdf]

S1 Table: Supporting information for Harvest plots

All results as reported in the included papers and the decision process underpinning the Harvest plot

Where outcomes within a category were conflicting, the decision process attached priority as follows:

- Defined primary outcomes in an adequately powered study
- Outcomes that measured impact in the whole eligible population (typically using routine data rather than data from a sub-group who accepted/completed the intervention or were recruited for the evaluation)
- Outcomes which were measured with a validated instrument (as opposed to responses to non-validated questions)
- Outcomes that were clinically as well as statistically significant (e.g. achieved s defined minimum clinically important difference)

Finally, if there were any remaining doubt, the authors' interpretation was considered as providing the context for our decision.

Abbreviations used in this table
Study Design: ITS: Interrupted time series RM: Repeated measures Long: Longitudinal QE: Quasi experimental B&A: Before and after
PAAP: Personalised asthma action plan HbA1c testing: Glycated haemoglobin testing QOF: Quality Outcome Framework

Citation design, size and quality	Reported outcomes<br>* indicates the primary outcome (if stated).	Researcher's interpretation for the Harvest plot
Beck 2004<br><br>QE<br>1 hospital, 16 paediatric patients who had an incident of DKA.<br>Quality score = 15	**Organisational process outcomes**<br>Programme participation<br>• Participants greater telephone contact (16 crisis management calls vs 0; p=.001)	Organisational processes and disease control both improved.<br><br>**Illustrated as positive effect**
	**Disease control**<br>Hospital admissions<br>• Decrease in hospital admissions from intervention group (1 emergency department visit or diabetic ketoacidosis episode vs 5 diabetic ketoacidosis hospitalisations; p=.039)	
	**Individual behaviour outcomes**	

	Not assessed	
Chien 2012<br><br>QE<br>118 practices, 5557 diabetes patients.<br>Quality score = 13	**Organisational process outcomes**<br>* Hba1C testing<br>Intervention group<br>• HbA1c testing : 2003 = 84% & 2004=85%, 2006 = 86% & 2007 = 91%<br><br>Control Group<br>• HbA1c testing : 2003 = 83% & 2004=85%, 2006 = 86% & 2007 = 87%	Diabetes care processes and outcomes did not improve significantly<br><br>**Illustrated as no effect**
	**Disease control**<br>HbA1c levels<br>Intervention group<br>• HbA1c <9b: 2003 = 36% & 2004 = 35%, 2006 = NA & 2007 = 32%<br><br>Control group<br>• HbA1c <9b: 2003 = 43% & 2004 = 38%, 2006 = NA & 2007 = 33%<br><br>The coefficient on intervention*post (difference in difference) was reported as not significant in these results, no p value provided.)	
	**Individual behaviour outcomes**<br>Not assessed	

Conrad 2013<br><br>QE<br>19 medical groups,<br>21,365 patients<br>Quality score = 10	**Organisational process outcomes**<br>Quality Incentive Programme<br>• regression results : 2003-04= -0.001 & 2005-07 = -0.04<br><br>Quality scorecard<br>• regression results: 2003-04 = -0.019 & 2005-07 = -0.004	no significant positive effect on general clinical quality<br><br>QIP 05-07 statistically significant negative result showing a reduction in quality<br><br>**Illustrated as negative effect**
	**Disease control**<br>Not assessed	
	**Individual behaviour outcomes**<br>Not assessed	
Fagan 2010<br><br>QE<br>20,943 65+ year old patients.<br>Quality score = 16	**Organisational process outcomes**<br>*HbA1c testing<br>• Intervention Group – Odds ratio = 1.66; 95%CI (1.14, 2.43)<br>• Comparison Group – Odds ratio = 3.76; 95%CI (3.42, 4.13)<br>• Intervention relative to Comparison – Odds ratio = 0.44; 95%CI (0.30, 0.65)	**Illustrated as no effect**
	**Disease control**<br>Not assessed	
	**Individual behaviour outcomes**<br>Not assessed	
Gulliford 2007<br><br>Long,	**Organisational process outcomes**<br>HbA1c testing<br>• HbA1c recorded in year: 2000 = 60, 2001 = 72, 2002 = 80, 2003 = 78, 2005 = 95	▪Increase in tests performance (until 2002)

26 general practices,<br>2099 patients.<br>Quality score = 17	**Disease control**<br>HbA1c levels<br>• HbA1c ≤7.4%: 2000 = 22, 2001 = 32, 2002 = 37, 2003 = 38, 2005 = 57<br>• HbA1c ≤10%: 2000 = 52, 2001 = 64, 2002 = 70, 2003 = 72, 2005 = 89<br><br>(No further statistics provided on these outcomes)	▪ increase in HbAc1 target of <7.4% & HbA1c <10%<br><br>**Illustrated as positive effect**
	**Individual behaviour outcomes**<br>Not assessed	
Kontopantelis 2012<br><br>ITS<br>148 practices, 23,920 patients.<br>Quality score = 17	**Organisational process outcomes**<br>HbA1c testing<br>• HbA1c recorded in year (SD): 2000/1 = 71.1 (45.3), 2001/2 = 77.9 (41.5), 2002/3 = 82.8 (37.7), 2003/4 = 89.2 (31.1), 2004/5 = 93.0 (25.5), 2005/6 = 93.7 (24.3), 2006/7 = 93.5 (24.6)	▪Increase in tests performance (until 2005/6)<br><br>▪Increase in HbAc1 target of ≤7.4%<br><br>▪Increase in HbAc1 target of ≤10% (until 2004/5).<br><br>**Illustrated as positive effect**
	**Disease control**<br>HbA1c levels<br>• HbA1c ≤7.4% (SD): 2000/1 = 45.5 (49.8), 2001/2 = 48.4 (50.0), 2002/3 = 50.2 (50.0), 2003/4 = 52.2 (50.0), 2004/5 = 55.6 (49.7), 2005/6 = 56.4 (49.6), 2006/7 = 59.3 (49.1)<br><br>• HbA1c ≤10% (SD): 2000/1 = 88.5 (31.9), 2001/2 = 90.4 (29.4), 2002/3 = 90.8 (28.9), 2003/4 = 91.8 (27.4), 2004/5 = 92.6 (26.3), 2005/6 = 92.5 (26.3), 2006/7 = 92.7 (26.0)	
	**Individual behaviour outcomes**<br>Not assessed	
LeBlanc 2017<br><br>Long<br>583 physicians, 83,580 adult patients	**Organisational Process outcomes**<br>HbA1c testing<br>• ≤2 HbA1c tests per year: univariate model OR = 1.16 (p<0.0001); 99%CI (1.11 1.20). Multivariate model OR = 1.23 (p<0.0001); 99%CI (1.18, 1.28)	**Illustrated as positive effect**

Quality score = 13		
	**Disease control**<br>HbA1c levels<br>• All patients: univariate model OR = 0.00; 99%CI (-0.03, 0.02).<br>Multivariate model OR = -0.01; 99%CI (-0.03, 0.02<br>• HbA1C 6.5% to 7.0%: univariate model OR = -0.02 ($p<0.0001$); 99%CI (-0.04, 0.01).<br>Multivariate model OR = -0.02 ($p<0.0001$); 99%CI (-0.04, 0.01).<br>• HbA1C 7.1% to 8.9%: univariate model OR = 0.03; 99%CI (-0.01, 0.08).<br>Multivariate model OR = 0.02; 99%CI (-0.02, 0.06).<br>• HbA1C ≥9%: univariate model OR = 0.04; 99%CI (-0.06, 0.15).<br>Multivariate model OR = 0.00; 99%CI (-0.10, 0.10)	▪No statistically significant changes in mean HbA1c levels<br><br>**Illustrated as no effect**
	**Individual behaviour outcomes**<br>Not assessed	
Mandel 2007<br><br>RM<br>44 paediatric practices<br>13 380 children.<br>Quality score = 16	**Organisational process outcomes**<br>Asthma action plan ownership.<br>• 19 (70%) achieved the 80% threshold for the PAAP.<br>• The cumulative percentage of the network all-payer asthma population receiving “perfect care” increased from 4% to 88%, with 18 of 44 practices (41%) achieving a perfect care percentage of 95% or greater<br><br>(no statistics reported)	**Illustrated as positive effect**
	**Disease control**<br>Not assessed	
	**Individual behaviour outcomes**<br>Not assessed	
Pape 2015	**Organisational process outcomes**	

B&A<br>1 primary care trust,<br>6,142 patients.<br>Quality score = 18	Not assessed	
	**Disease control**<br>HbA1c levels<br>HbA1c of ≤8% :<br>• Exception reporting Baseline = 0.085, Secular trend effect = 0.001 (p = 0.910), QOF+ baseline = 0.060 (p=0.018)<br>• Controlled Patients Baseline = 0.725, Secular trend effect = 0.015 (p=0.005), QOF+ baseline = 0.002 (p=0.968)<br><br>HbA1c of ≤9% :<br>• Exception reporting Baseline = 0.062, Secular trend effect = 0.001 (p = 0.891), QOF+ baseline = 0.043 (p=0.049)<br>• Controlled Patients Baseline = 0.822, Secular trend effect = 0.015 (p=0.002), QOF+ baseline = 0.003 (p=0.934)	▪No statistically significant improvements in mean HbA1c levels<br><br>▪ Increase can be attributed to increase in exception reporting since intro of QOF+<br><br>**Illustrated as no effect**
	**Individual behaviour outcomes**<br>Not assessed	
Rosenthal 2005<br><br>QE<br>205 physician groups,<br>1,174,294 patients.<br>Quality score = 18	**Organisational process outcomes**<br>HbA1c testing<br>Intervention group<br>• Pre Quality Incentive Programme - 62.0%, after QIP 64.1%,<br>• Difference (Post-pre), 2.1% (SE 1.0)<br>• P value .02<br><br>Control group<br>• Pre Quality Incentive Programme - 62.0%, after QIP 64.1%,<br>• Difference (Post-pre), 2.1% (SE 1.0)<br>• P value .02	▪Slight improvement but not significantly different from comparison group<br><br>**Illustrated as no effect**

	**Disease control**<br>Not assessed	
	**Individual behaviour outcomes**<br>Not assessed	
Vamos 2011<br><br>ITS,<br>422 general practices<br>154 945 patients.<br>Quality score = 15	**Organisational process outcomes**<br>• HbA1c measured (95% CI)- 1997, by quintile: 32.8 (31.8-33.7), 31.2 (30.2-32.0), 34.6 (33.7-35.6), 32.2 (31.2-33.0), 37.7 (36.7-38.7)<br>• HbA1c measured (95% CI)- 2005, by quintile: 74.0 (73.4-74.6), 76.4 (75.8-76.9), 77.3 (76.7-77.8), 73.9 (73.3-74.5), 76.2 (75.6-76.8)	
	**Disease control**<br>HbA1c mean levels<br>• HbA1c mean (95% CI)- 1997, by quintile, 7.6 (7.5-7.7), 7.6 (7.5-7.7), 7.7 (7.6-7.8), 7.5 (7.4-7.6), 8.2 (8.1-8.3)<br>• HbA1c mean (95% CI)- 2005, by quintile, 7.5 (7.5-7.5), 7.4 (7.4-7.4), 7.4 (7.4-7.4), 7.5 (7.4-7.5), 7.4 (7.4-7.5)<br>• Baseline proportion of patients meeting HbA1c <7.0% in 1997: 35.3, 95% CI = 31.0-39.7, p<0.05<br>• Annual change before introduction of P4P: 2.0,95% CI = 1.3-2.7, p<0.05<br>• Annual change in the year P$P introduced: 0.8, 95% CI = -1.8-3.5,<br>• Annual change after P4P was introduced: -2.2, 95% CI = -4.0- -0.4, p<0.01	▪ No significant additional improvement<br><br>**Illustrated as no effect**
	**Individual behaviour outcomes**<br>Not assessed	
Young 2007	**Organisational process outcomes**<br>HbA1c testing	▪ No difference between post & pre-intervention trends.

ITS,<br>334 Primary care physicians, unknown number of patients.<br>Quality score = 16	• Adherence rates: mean (SD) pre-intervention: 1999 = 0.56 (0.23), 2000 = 0.57 (0.19), 2001 = 0.59 (0.17)<br>• Adherence rates: mean (SD) post-intervention: 2002 = 0.62 (0.17), 2003 = 0.61 (0.18), 2004 = 0.63 (0.18)<br>• Change in adherence rate: 2000-2001 = 0.018; 2001-2002= 0.026, p<.05<br>• Difference in rate of change (2001-2000)(vs (2002-2004) = 0.009 (no p value given)	▪ Overall increase in performance result of secular trends<br><br>**Illustrated as no effect**
	**Disease control**<br>Not assessed	
	**Individual behaviour outcomes**<br>Not assessed	
