# Supplementary figures and images for: The impact of financial incentives on the implementation of asthma or diabetes self-management: A systematic review
Source: PLoS One. 2017 Nov 6;12(11):e0187478. doi: 10.1371/journal.pone.0187478 (PMC5673190; doi:10.1371/journal.pone.0187478)

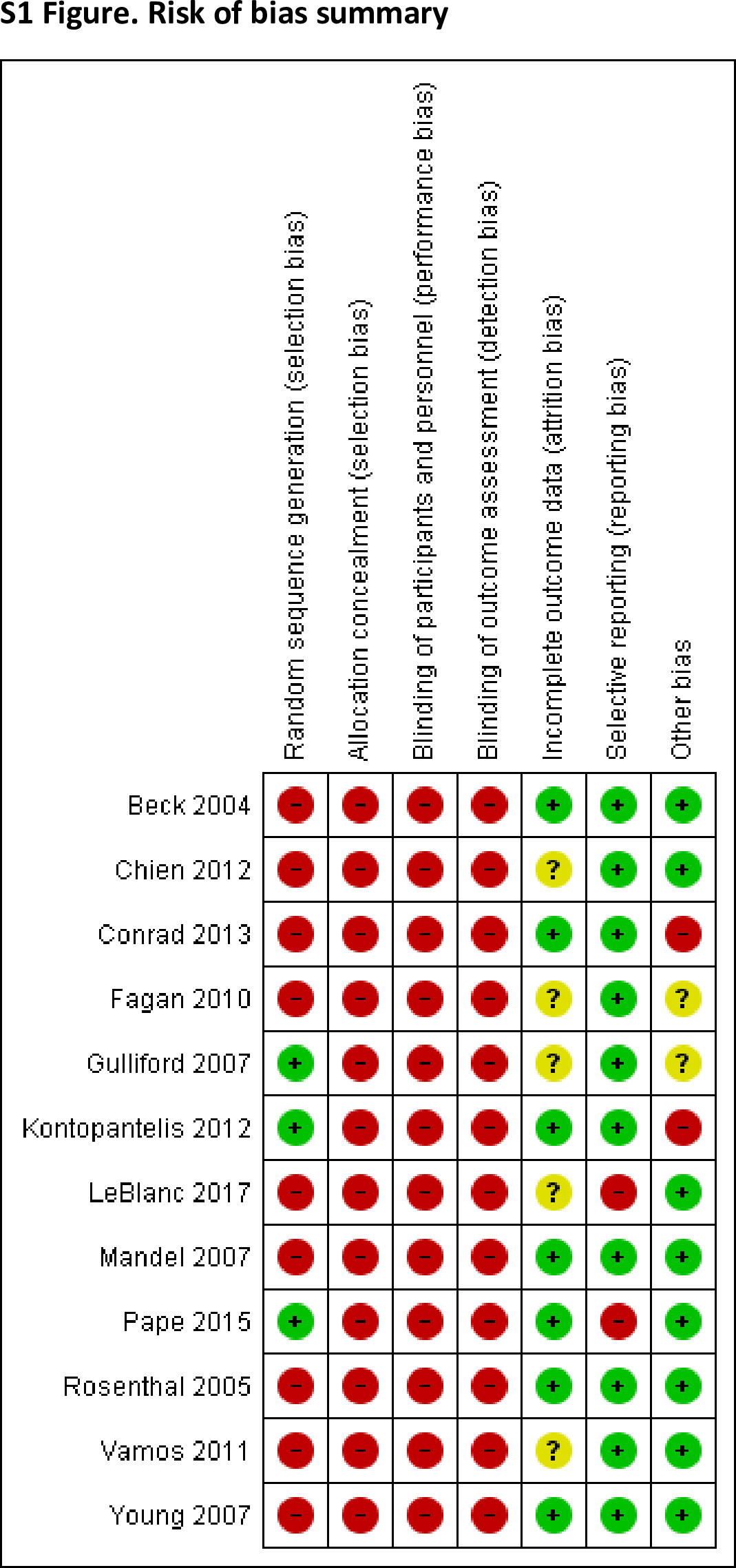

Supplement: S1 Fig — (TIF) [file pone.0187478.s005.tif]
